# Resin Chemistry-Driven Starch Reinforcement in Water-Free Photocurable Networks

**DOI:** 10.1021/acsomega.6c02042

**Published:** 2026-06-24

**Authors:** Md Arif Mahmud, Chamil Abeykoon, Prasad Potluri, Anura Fernando

**Affiliations:** Department of Materials, School of Natural Sciences, Faculty of Science and Engineering, 5292 University of Manchester, Manchester M13 9PL, U.K.

## Abstract

Starch was added to water-free photocurable systems comprising biocompatible polymers (e.g., polyols and carbonates) in combination with methacrylic acid-based polymers to provide reinforcement despite its compatibility and processing challenges. Mechanical, thermal, FTIR, SEM, DMA, PSI, and gel fraction analyses were performed as functions of different combinations. The all-methacrylate formulation produced the highest tensile strength (17.0 MPa with 40% starch loading, along with a flexural strength of 13.8 MPa), while the highest flexural strength (17.1 MPa) was reported at lower starch loading with reduced tensile performance, indicating stiffening at the expense of toughness. Conversely, combinations involving biocompatible polymers, as well as the use of coupling agents, resulted in lower mechanical performance, as DMA and gel fraction results indicate more rigid network behavior in methacrylate-based systems, whereas more starch-compatible systems showed increased network heterogeneity and reduced uniformity, which demonstrated the influence of resin chemistry on starch incorporation in such systems.

## Graphical abstract

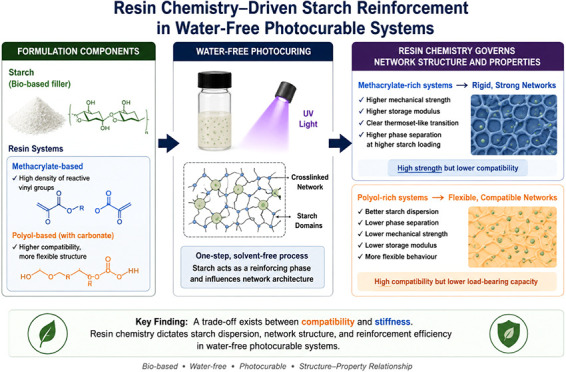

## 1 Introduction

Synthetic polymers are used in various applications in all sectors, which have become a concern due to the source of the polymer, the synthesizing process, and the lifecycle.^1^ These polymers include cellulose and starch, which are polysaccharide-based, derived from vegetable sources, and keratin, a protein-based polymer derived from animal sources.^2–4^ Among these polymers, starch has been proposed as a solution because of its availability in abundance and its advantages in terms of eco-friendliness and biocompatibility. However, their properties are far from matching the requirements set by many synthetic polymers and need to undergo chemical or physical modifications from lower to higher degrees. In this context, ester bond formation or ester linkage is one of the most common chemical modification treatments applied to biopolymers. An ester link is formed between the hydroxyl (OH) of biopolymers and the carboxyl (COOH) groups, helped by chemical or thermal catalysts and coupling agents.^5^ Because of the presence of the OH groups, natural polymers readily form ester linkages, which are among the most common chemical bonds associated with them. This also promotes compatibility with polymers containing OH and COOH functionalities. Esterification further allows control over hydrophilicity, leading to improved mechanical and thermal performance without compromising the biocompatibility.^6,7^

Natural polymers are considered the most attractive and sustainable option by many; however, aqueous processing, multistep modification, and energy-intensive conditions, with filtration and significant material loss,^8–13^ often make natural polymers less sustainable. Furthermore, natural polymers are usually employed as nonreactive fillers rather than active structural components, while photocuring is a fast, low-energy, and efficient pathway to material fabrication.^14–19^ However, the direct application of natural polymers in photocurable systems is limited by their lack of intrinsic photoreactivity as well. On the other hand, one attractive alternative to reduce processing steps and material loss could be an integrated processing approach where chemical reactions and material formation are performed in the same medium.^8,20,21^ However, applying such approaches to natural polymers remains a challenge because of the required aqueous environments that are often unavoidable for natural polymers. Another limitation of this system is the selection of the processing medium, as conventional solvents such as dimethyl sulfoxide (DMSO) and dimethylformamide (DMF) may facilitate dissolution and reaction, but they cannot be part of the final material and must be removed after processing. The use of a reactive medium, where the solvent can act as both reaction environment and structural component of the final polymer network, could be tunable eutectic-type systems, comprising two or more compounds where their components exhibit melting point depression and multifunctional behavior, potentially has the ability to allow all chemical interactions while also contributing to network formation. The concept of polymerizable deep eutectic systems has been explored in previous studies,^22^ and it has been shown that appropriately designed mixtures can allow for water-free and solvent-free processing. Thus, the development of water-free photocurable systems based on such reactive media presents a potential route for incorporating natural polymers into structurally functional networks without multistep processing.

Under these conditions, the use of starch in water-free photocurable systems is limited, and in most of the works reported, it is used as a nonreactive filler that is processed through aqueous or multistep modification routes, which limits its structural contribution and processing efficiency. In this work, starch is employed as a biobased component that serves as the reinforcing phase and also contributes to the structural behavior of the resin system; the formulation includes methacrylate-based components and other low-toxicity additives, with methacrylic acid and its derivatives chosen to impart photocurable functionality (while acrylic acid is more photoreactive and is typically employed in such systems, the high reactivity of acrylic acid can make it difficult to control, so a methacrylic acid-based system offers more controllable reactivity and lower risks of homopolymerization, as well as improved environmental compatibility).^23–25^

The goal of this study is to clarify the impact of the resin composition on the integration of starch and to correlate these effects with the mechanical, thermal, viscoelastic, and morphological properties of the developed composite structures. This work addresses this limitation by designing a water-free photocurable system where starch has been incorporated as a functional component through resin chemistry rather than as a passive filler. As a result, the study is structured around a central structure–property question: how does resin chemistry determine whether starch behaves as a source of network heterogeneity or as a reinforcing phase? The study has been structured around representative formulations that are selected to highlight the key structure–property relationships that govern starch behavior in water-free photocurable systems.

## 2 Materials and Methods

The materials intended for material development are as follows.

Soluble starch has been selected as the main biobased reinforcing polymer option. Starch (molecular weight, Mw = 342 g/mol) and glycerol were purchased from APC Pure U.K. Citric acid (CA anhydrous, Mw = 192.12 g/mol) and methacrylic acid (MAA, Mw = 86.09 g/mol) were purchased from Fisher Scientific. Ethylene glycol-based methacrylate (2-hydroxyethyl methacrylate: EGMA, Mw = 130.14 g/mol), triethanolamine (TEOA), low-molecular-weight poly­(ethylene glycol) dimethacrylate (PEGDMA, Mw = 550 g/mol), aliphatic polycarbonate (APC: propylene carbonate), epoxy-terminated poly­(ethylene glycol) coupling agent (poly­(ethylene glycol) diglycidyl ether: EPEGC, Mw = 500 g/mol) for ether formation, poly­(ethylene glycol) (PEG 400, Mw = 400 g/mol), and ethanol were purchased from Sigma Aldrich U.K. Carbodiimide coupling (CIC) agent (EDC·HCl, Mw = 191.70 g/mol) for ester formation and photoinitiator Igracure 819 (PI) were purchased from Apollo Scientific U.K., and a nonionic surfactant (Polysorbate 80) was purchased from Amazon U.K., which was to be used only to prevent phase separation of starch. For photocuring, a 405 nm wavelength UV curing unit was used, and for material processing, a hotplate magnetic stirrer, thermometer, and other basic chemistry laboratory equipment were used for material development.

The materials have separate functions in the process, which are stated below.

- Main polymers: The backbone of the structures consists of a reactive medium, of which starch acts both as a physical reinforcement and as a part of the chemical structure by reacting with the reactive ends of the main resin and forming bonds. In this work, citric acid (CA), glycerol, poly­(ethylene glycol) (PEG 400), methacrylic acid (MAA) and its different derivatives (EGMA, PEGDMA), APC, and TEOA are selected to form the reactive medium or solvent, which will also act as the photopolymerizable or photocurable parts of the system. The additional parts are essential for developing photocurable components because of the absence of photocurable ends in starch. In addition, PEGDMA can also work as the photocross-linking agent, as well as a diluent for these mixtures.
- Modifying chemicals: CIC and EPEGC have been selected for this work, which are based on ester and ether bond formation, as the modifying chemicals for starch. The selections have been made based on previous studies. Apart from the alkaline or acidic conditions, no other additional catalysts are required for these modifying chemicals.

The selected materials for this study contain biobased or biodegradable polymers such as starch, CA, APC, etc. However, acrylic-based polymers are often considered toxic, while methacrylate-based compounds such as MAA, EGMA, and PEGDMA are considered to have lower toxicity and are often used in the medical sector.^26–33^ These are not considered toxic because they are hydrophilic, require mild UV for processing, are more cytocompatible, and are less aggressive than acrylic-based compounds; however, they are not biodegradable after curing.^26,27,33–35^ The coupling agents selected here are CIC and EPEGC, where CIC forms urea-based byproducts that are not highly toxic and can act as a plasticizer for composites, while EPEGC is known as a plasticizing cross-linking agent that can be associated with biobased polymers.^36–39^ Therefore, these materials align with the objective of this study, i.e., developing lower-toxicity photocurable composite structures.

### 2.1 Resin Preparation

**Set A** of the samples was prepared by EGMA, MAA, and PEGDMA combined with starch loading and binding. It is assumed that the mixture is bonded initially by hydrogen bonding between the compounds, with hydrogen bonding resulting from the donation of protons from the free carboxyl (COOH) of MAA and the hydroxyl (OH) of EGMA and from the addition of the hydroxyl (OH) of the ester bonds of EGMA and the carbonyl (COOH) of MAA and the starch. The mixture helps to dilute and partially homogenize the starch, which should be sufficient to allow the surface hydroxyl groups to be more readily available for bonding with other reactive ends of the mixture. Then, the starch was mixed for several hours at its gelatin temperature^5^ before the samples were prepared by adding PI and photocuring for 5–10 min first and then again for 30 min after a gentle washing with photocuring resin detergents. When EPEGC was used, an additional hour at almost 80 °C was used to complete the process, whereas CIC only required an hour at about 50 °C. The samples were initiated with a mixture in which MAA was the predominant percentage, and EGMA was used as a stabilizing component to allow the incorporation of starch and its hydroxyl group into the system, which makes it hydrophilic in nature. Starch is distributed in the EGMA–MAA mixture, where MAA not only provides hydrogen bonds in the network that help to expand the starch by opening the structure by breaking the chain but also prevents the formation of intermolecular hydrogen bonds in the starch, thereby preventing its degradation. In addition, this combination helps starch to integrate into a network of hydrophilic, hydrogen-bond-rich structures, which allows it to be homogenized.^40^ The percentage of EGMA, MAA, and PEGDMA was changed to find the optimal state for the incorporation of starch in the system, while the latter part used various CIC and EPEGC bonding agents to form the network bonds.

**Sets B**–**D** were based on the classical deep eutectic solvent systems of polyols (B and C) and polyols (D), which are well-known for natural polymer processing.^41,42^ An eutectic mixture is a mixture in which the melting points of the individual components are reduced from their original values. This depression occurs because of molecular interactions between the components, generally via hydrogen or ionic bonding, disrupting the structure of each component. The most widely studied eutectic mixture is deep eutectic solvent (DES), which is a fully hydrogen-bond-based system formed using a hydrogen bond donor (HBD) and a hydrogen bond acceptor (HBA), often stabilized by hydrogen bonding and/or ionic bonding, while a significant depression takes place in the melting temperature of the compounds. The HBDs generally have a hydrogen atom attached to an electronegative atom (O, N; for example, OH, COOH, NH), while the HBAs contain an electronegative atom (O, N) with lone pairs of electrons that can accept a hydrogen bond. In this way, functional groups such as hydroxyl (OH), carboxyl (COOH), amino (NH_2_), and amide (CONH_2_), which are preferentially all HBDs, can actually play both roles of HBD or HBA depending on the compound with which they are paired. Other groups like carbonyl (CO), ether (C–O–C), and tertiary amine (NR3) are only capable of functioning as HBAs.^43,44^ The combinations used in B, C, and D are CA:PEG 400 (2:1), CA:glycerol (1:2), and glycerol:APC (1:1), all of which are known for their HBD and HBA functions, as CA and glycerol play the role of HBDs, while PEG 400 and APC serve as HBAs.^45–48^ The combinations were prepared by mixing the components and heating them until they formed a homogeneous liquid. Then, the starch was added, and the heat was switched on. For CA systems, heating was carried out at 160–170 °C for 2 h. Then, trace TEOA was added to slightly increase the pH of the solution, followed by refrigeration and addition of MAA with a CIC, which is best for acidic conditions at a specific pH.^49^ In the case of CIC, the neutralization was only completed at the end of its own process. The APC was also used as a compatibilizer where appropriate. For set D, both glycerol and APC were liquid, and the melting temperature drop in this system, similar to those of sets B and C, was not apparent, remaining below 100 °C from 150 °C. However, taking these compounds as HBDs and HBAs, respectively, the mixture can be classified as a eutectic mixture and is expected to be effective in dispersing the reactive starch. The same procedure used in Set A was followed to obtain the final sample by using coupling agents and PI.

### 2.2 Composite Structure Development by Photocuring

This step is for developing the photocurable resin that can be photocured to produce a composite structure. Both types of solutions were prepared using a similar process to make the final material. PI was mixed with the developed resins, followed by pouring into transparent glass molds and then placing in the photocuring unit for 10 min initially, followed by another 30 min for full curing. The produced samples were then left in the laboratory environment for conditioning and any further self-curing for a day, while the samples were washed with ethanol to remove residual uncured resin and wiped properly before being left in the room for conditioning. The EPEGC-containing samples were oven-dried at 80 °C for 20 min after photocuring to complete the epoxy-mediated coupling.

The samples with different combinations are listed in Table 1. The prepared samples were then taken for different tests for characterization.

**Table 1:** List of Samples

			main resin (W/W)		additives (W/V on the weight of Main Resin)		
			base components %	cross-linker %		coupling agent	
name of the set	assessment parameter	sample name	eutectic mixtures	PEGDMA	starch %	EPEGC %	CIC %
Set A: MAA–EGMA mix	Variable Studied on MAA–PEGDMA: SA1–SA4: MAA varied from 50% to 20%, while PEGDMA varied from 10% to 40%	SA1	90	10			
		SA2	80	20			
		SA3	70	30			
		SA4	60	40			
	SA5: 30% MAA and 30% EGMA comprised the 60% base component	SA5	60	40			
	Variable studied on starch loading against SA4 (noncoupled): starch was loaded from 10% to 40%	SA6	60	40	10		
		SA7	60	40	20		
		SA8	60	40	30		
		SA9	60	40	40		
	Variable studied on starch loading against noncoupled (coupled): starch was loaded and coupled with CIC	SA10	60	40	10		10
		SA11	60	40	20		10
		SA12	60	40	30		10
		SA13	60	40	40		10
	Variable studied on different coupling agents: Fixed starch loading was associated with variable quantities of coupling agents (CIC and EPEGC)	SA14	60	40	30^a^		15
		SA15	60	40	30^a^		20
		SA16	60	40	30^a^	10	
		SA17	60	40	30^a^	15	
		SA18	60	40	30^a^	20	
Set B: CA–PEG 400 mix	Variable studied on starch loading (coupled) against different mixes: starch loading varied from 0% to 30% while the coupling agent CIC remained constant for Set B and Set D.Only 0% and 30% starch loading were used for Set C samples	SB1	60	40			
		SB2	60	40	10		10
		SB3	60	40	20		10
		SB4	60	40	30		10
Set C: CA–glycerol mix		SC1	60	40			
		SC2	60	40	30^a^		10
Set D: Glycerol–PC Mix		SD1	60	40			
		SD2	60	40	10		10
		SD3	60	40	20		10
		SD4	60	40	30		10

a 30% starch loading was taken as constant because of increasing variability in properties in samples containing high starch loading (40%).

Here, Table 1 presents the compositions of resin systems and experimental variables. Set A examines cross-linker ratio, starch loading, and coupling effects. Sets B–D explore composition-dependent behavior under similar conditions.

### 2.3 Testing of Resin and Prepared Samples

Testing was conducted in two phases. One phase was carried out with the resins in the resin form, while the other phase was conducted after the resins were photocured. The tests that were conducted are discussed as follows.

#### 2.3.1 Resin Density Testing

The resin density test was conducted according to the method of ASTM D1475. A known mass of the resin was placed in a measuring plate, and the volume of the resin was measured from the plate’s reading. The density was then calculated by using the following formula.


1 density=massing/volumeinccg/cc

#### 2.3.2 Resin Phase Separation Index Testing

The phase separation index (PSI) test was carried out using the procedure of the ASTM D869 method. Specific masses of resins were left undisturbed for 24 h at room temperature in beakers of specific volume. Then, after the specified duration, the height of the settled part and the height of the separated part were measured, and the PSI percentage was calculated using the following equation.

2 $$p\mathrm{hase}\;\mathrm{separation}\;\mathrm{index}\;\left(\mathrm{PSI}\%\right)=\left(H_{\mathrm{separated}‐\mathrm{layer}}\right)/\left(H_{\mathrm{total}}\right)\times 100$$

where

(*H*_total_) = original height of the sample in the tube.

(*H*_separated‑layer_) = height of the phase-separated layer at the top after the aging period.

#### 2.3.3 Tensile Test of the Prepared Sample

Tensile tests provide a set of results that include the tensile strength and elongation properties. The tensile strength is a measure of the required load to break a material, while the extensibility is an indicator of the flexibility and elongation properties of the material, measured from its displacement from the initial length before breaking.^50,51^ The tensile test was conducted using an Instron Universal Tensile Tester, while the test method for the specimen was ISO 527–2 5A, and the crosshead speed was 4 mm/min.

#### 2.3.4 Flexural Resistance of the Prepared Sample

The ASTM D790 standard was utilized to assess the flexural properties of the samples using the 3-point bending test. Rectangular specimens of specific dimensions (16:1 span-to-depth ratio, support span:specimen thickness) were tested on a three-point bending system in a universal testing machine fitted with a 500 N load cell. The crosshead speed was set to 10 mm/min, according to the standard. The procedure was used to determine the maximum stress at the outer surface of the specimen at the moment of failure, which is the flexural resistance of the material, and the flexural modulus was determined from the slope of the initial linear portion of the load–deflection curve. All of the specimens were tested and compared for the assessment.

#### 2.3.5 Hardness of the Prepared Sample

The hardness of the prepared samples was assessed using the ASTM D2240 durometer hardness. This test method determines the indentation hardness of materials by measuring the resistance of the material to penetration of a specific indenter under a defined force. Samples were tested using a durometer hardness tester fitted with a type D indenter, appropriate for materials with shore D hardness values. The specimens were made to a thickness of 3 mm by stacking a couple of samples having flat and uniform surfaces. The test was carried out by bringing the indenter tip into contact with the surface perpendicularly and applying a firm, even pressure without a shock. Readings were taken at different locations on the surface, and the average Shore D hardness results were reported, along with the standard deviation.

#### 2.3.6 Dynamic Mechanical Analysis (DMA)

Dynamic mechanical analysis (DMA) was conducted using TA Instruments Q800 in the dual cantilever mode. Rectangular specimens (35 mm span) were tested under strain-controlled oscillation. For this study, temperature sweeps were performed, and the range was from 25 to 140 °C with a soak time of 2 min, a fixed frequency of 1 Hz, and a ramp rate of 3 °C/min. The storage modulus (*E*′), loss modulus (*E*″), and damping factor (tan δ) were recorded as functions of temperature. The glass transition temperature (*T*_g_), along with the homogeneity and elastic/plastic nature of the material, was determined from the results.

#### 2.3.7 Gel Fraction Test

Gel fraction is defined as the percentage of insoluble polymer left in a material after solvent extraction (e.g., ethanol). This represents the cross-linking density in the material as well as the solubility. The test was carried out according to the ASTM D2765 standard. The samples were oven-dried to remove any moisture and weighed (*M*_0_). Then, the samples were placed in ethanol and kept in the solvent for 72 h. Then, the samples were oven-dried and weighed (*M*_D_) again. The gel fraction was calculated from eq 3


$$g\mathrm{el}\;\mathrm{fraction}\%=M_{D}/M_{0}\times 100$$ 3

#### 2.3.8 Thermogravimetric Analysis (TGA) of the Prepared Sample

Thermogravimetric analysis (TGA) was done to evaluate the thermal stability and degradation behavior of the composite structures after curing. Measurements were performed using a thermogravimetric analyzer TGA Q500 V20. About 5–10 mg of each sample was placed in a platinum crucible and heated from room temperature to 600 °C at a constant heating rate of 10 °C min^–1^ under a continuous flow of nitrogen atmosphere. The onset degradation temperature and weight-loss profiles were measured from the resulting thermograms produced by the machine. The temperatures corresponding to 5% (*T*_5_), 10% (*T*_10_), and 50% (*T*_50_) weight loss were determined from the TGA curves. The residual mass at 600 °C was recorded as char or residue to assess the percentage of char formation and thermal residue.

#### 2.3.9 Fourier Transform Infrared Spectroscopy (FTIR)

FTIR spectra of the raw materials and the composite structures were recorded using a Bruker α spectrometer in the range between 400 and 4000 cm^–1^. Characteristic peaks within these regions were detected. Interpretation of the peaks would show the newly formed bonds, changes in any bonds, and stretching and vibration of the functional groups, which would indicate the possible bonding among the compounds of the mix. The machine was capable of identifying both absorbance and transmittance data, although the absorbance results would be considered in this work.

#### 2.3.10 Scanning Electron Microscopy (SEM) Analysis

The Quanta 650 SEM instrument was used to assess the morphological features of the samples. Images were taken from both the surface and the cross-sectional area for different samples. The instrument was operated under vacuum with a spot size of 3.5 and a voltage of 5 kV. The samples were first coated with a 5nm gold–palladium (AuPd) coating using a vacuum sputter coater to enhance image quality. The samples were mounted on aluminum stubs using conductive carbon tape, and then a silver side coating was applied to further improve electron conductivity. Then, the samples were loaded into the SEM chamber. Imaging was carried out using specific magnifications (1000×) for the assessment.

## 3 Results and Discussion

Test results for different properties of the samples are shown in this section. All these are average results where the number of samples for each result is 3 (*n* = 3), and error bars in the figures are the standard deviations.

The results are discussed and explained in the following sections.

### 3.1 Density and PSI% of the Resins of Different Combinations

The densities and PSI% of different resins were measured, and the trends found from those describing the impact of starch loading are presented in Figure 1.

**Figure 1:**
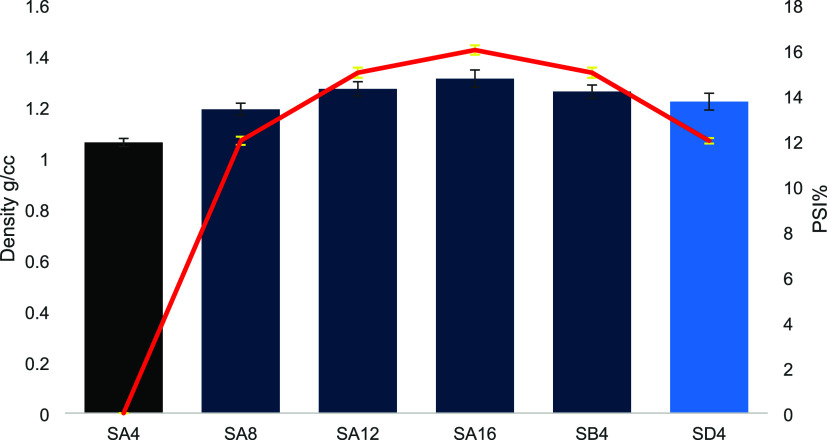
Density and PSI% of resin systems as a function of starch loading (with 10% CIC and 10% EPEGC) in different combinations. Both increase with increasing starch content, indicating a higher density of starch and a decrease in free volume within the network, along with reduced compatibility and increased phase separation at higher filler loading. The decrease in PSI% was reported when better compatible systems were used in polyol-based systems (Set B and Set D).

Figure 1 shows the effect of the starch loading on the density of the resins. The results from a gradual increase in starch loading in different systems indicate an increase in the density of the resin with increasing amount of starch for all combinations. The lowest density was observed in the EGMA–MAA–PEGDMA-based sample at approximately 1.05 g/cm^3^, but it gradually increased in all combinations with a gradual increase in the percentage of starch due to higher starch content (about 1.5 g/cm^3^),^52^ which adds to the densities of the resins as shown in Figure 2.^53^ The density of the CIC-treated resins is also increased by potentially adding a coupling agent byproduct, making the structure more compact. As a result, the density increased,^54^ although the difference is very small compared to samples containing no CIC. Another bonding agent, EPEGC (ether binding agent), has the possibility of forming a slightly higher degree of cross-linking than CIC due to having two epoxide groups per chain, as opposed to one ester bond per CIC molecule. This presents the potential of forming denser structures.^55,56^

**Figure 2:**
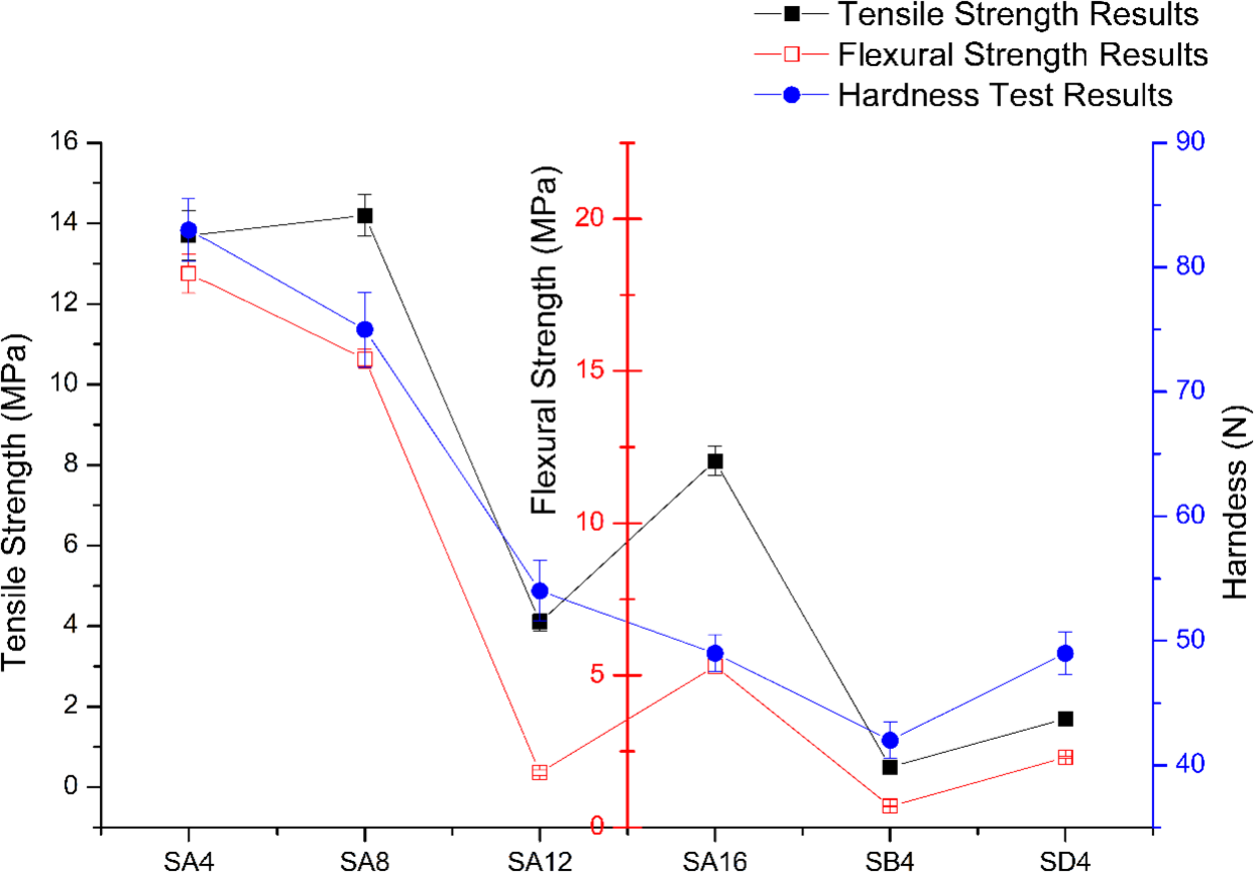
Effect of starch loading and coupling agent concentration (CIC and EPEGC) on tensile and flexural strength and hardness property against starch loading (30% w/v) and coupling in different systems. Tensile strength increased with starch loading but decreased with coupling agent content, which was common in flexural and hardness tests, suggesting that coupling introduced structural heterogeneity in the resin and made it softer than usual.

Again, Figure 1 also shows that PSI increases with increasing starch content due to the increased viscosity of the starch-containing part of the resins. The PSI mechanism is highly dependent on the hydrophobic and hydrophilic properties of the material, as the addition of highly hydrophilic starch in large quantities separated the less hydrophilic and less hydrophobic parts of the material and resulted in the aggregation of particulates, leading to a higher PSI. The high degree of hydrogen bonding between the components also caused the starch to bind more strongly to certain surfaces, resulting in phase separation.^57,58^ On the other hand, Sets B–D, based on polyols, showed less phase separation due to higher starch gelatinization and homogenization with controlled viscosity of the resins.^59^ However, EPEGC is plausibly more miscible with hydroxyl groups but still shows greater phase separation in Set A due to acidic conditions in Set A that render EPEGC unstable and result in local binding.

The combination of density and PSI data shows that even though the incorporation of starch improves the compactness of the material, it also lowers phase stability under high loads, underlining the delicate balance between reinforcement and compatibility. Lower PSI values for polyol-based resins indicate better dispersion and interaction with starch, while higher PSI values for methacrylate systems indicate less compatibility, despite their higher mechanical properties, as a result of the influence of the chemical in the resin on phase behavior and structural integrity.

### 3.2 Tensile and Flexural Properties of Different Photocured Samples

The tensile and flexural strengths and hardnesses of different samples are shown in Figure 2.

Representative formulations were selected to highlight the main formulation effects, and the mechanical performance was interpreted using these formulations rather than treated as isolated formulation trends. The trend shown by the effect of the starch loading on the tensile and flexural strengths of the samples studied shows that the tensile strength was increased and the flexural strength was decreased by the loading of the starch. This trend was observed for all combinations and samples with or without a coupling device. The starch particles were fillers in all these combinations^60^ and therefore increased the tensile strength, but their presence led to the agglomeration of partially gelatinized starch particles, which reduced the load transfer of the structures, decreasing the flexural strength.^61–65^

Meanwhile, the cross-linking agent increases tensile and flexural strength, indicating improved network rigidity. It has resulted in an increase in binding strength from 8.1 to 13.7 MPa as the cross-linker quantity increased from 10% to 40%.^66–68^ The same trend was observed in flexural strength as well. The trend of improvement was similar for samples from SA1 to SA4, but SA5 showed a decrease in both tensile and flexural strength, even though the quantity of PEGDMA, the cross-linker, remained the same. It happened because EGMA integrated flexible ends along with its methacrylate part, while MAA introduces a more polar and acidic portion in the structure with lesser flexibility. MAA’s hydrogen-bonding ability, along with its acidity, has the potential to disturb the structure and cause more heterogeneity. Meanwhile, the decreased uniformity probably also impacted stress distribution under bending, leading to a steeper decrease in flexural performance.^69,70^

However, the effect of coupling agents on Set A samples revealed that the addition of CIC significantly reduced tensile strength when compared to noncoupled samples with starch, and flexural strength also showed a similar pattern. According to PSI results, increased phase separation and structural heterogeneity are associated with strength loss. Additionally, the coupling agents in this system appear to interfere with the material’s uniform stress transfer, which lowers the mechanical performance. Although not as sharp as CIC, EPEGC exhibited the same declining trend in tensile and flexural strength. This implies that while coupling agents affect network interactions, mechanical strength is not generally improved by their influence in this system.^71,72^ Even though it creates new covalent bonds, the reduction is primarily caused by the above-described effect on the acidic ends, which leads to the formation of both ester and ether bonds. The chemistry of the resins is also impacted by decreased acidity, and as a result, starch begins to phase out. However, due to its better compatibility with starch, lack of byproducts such as CIC, and compatibility with the hydrophilic phase, its impact was less noticeable.^73–75^

Furthermore, hardness results indicate that as starch loading increased, the hardness first decreased and then increased. This is because relatively soft starch was incorporated into the matrix, resulting in the formation of a more rigid composite structure. This is particularly evident in noncoupled Set A samples, which exhibit a slight decrease in hardness, followed by an increase with increasing starch content. Coupling agents also decreased hardness, indicating greater structural flexibility; polyol-based sets (B and C) had lower hardness values, while Set A, with higher starch loading and no coupling agents, had the highest hardness.^76–78^ Therefore, in terms of creating hard materials, Set A without coupling and with higher starch loading produced better results. These findings imply that the developed composites’ balance between rigidity and flexibility is influenced by both filler loading and resin chemistry.

In general, Set A had the highest mechanical performance, followed by Set D and then Sets B and C, which were comparatively weaker. This trend is largely determined by the density of photocurable functional groups, with a higher concentration of reactive vinyl groups, leading to a stiffer and load-bearing network.^79^ Although Set D with glycerol and APC showed better results than Sets B and C with polyols due to the compatibility of the ingredients, there are several reasons for this trend. On paper, polyols of citric acid (CA) showed great promise with a certain ability to form covalent bonds during processing and improved compatibility with starch. In reality, however, the mixture of CA and polyols formed esters, but these esters behaved more like plasticizers than as plasticizers. On the other hand, the APC-based system has shown better results due to the high compatibility of APC with starch and its ability to disperse and limit starch agglomeration, resulting in a more homogeneous mixture.^80–87^ Therefore, mechanical performance is governed by the combination of cross-link density and structural homogeneity, and these trends are confirmed by the DMA and gel fraction results, which give information on the network structure and effective cross-link density.

### 3.3 Analysis of the DMA Test Results

DMA tests were performed on several samples through temperature sweep, and the storage modulus, loss modulus, and tan delta results are shown in Table 2 and Figure 3.

**Figure 3:**
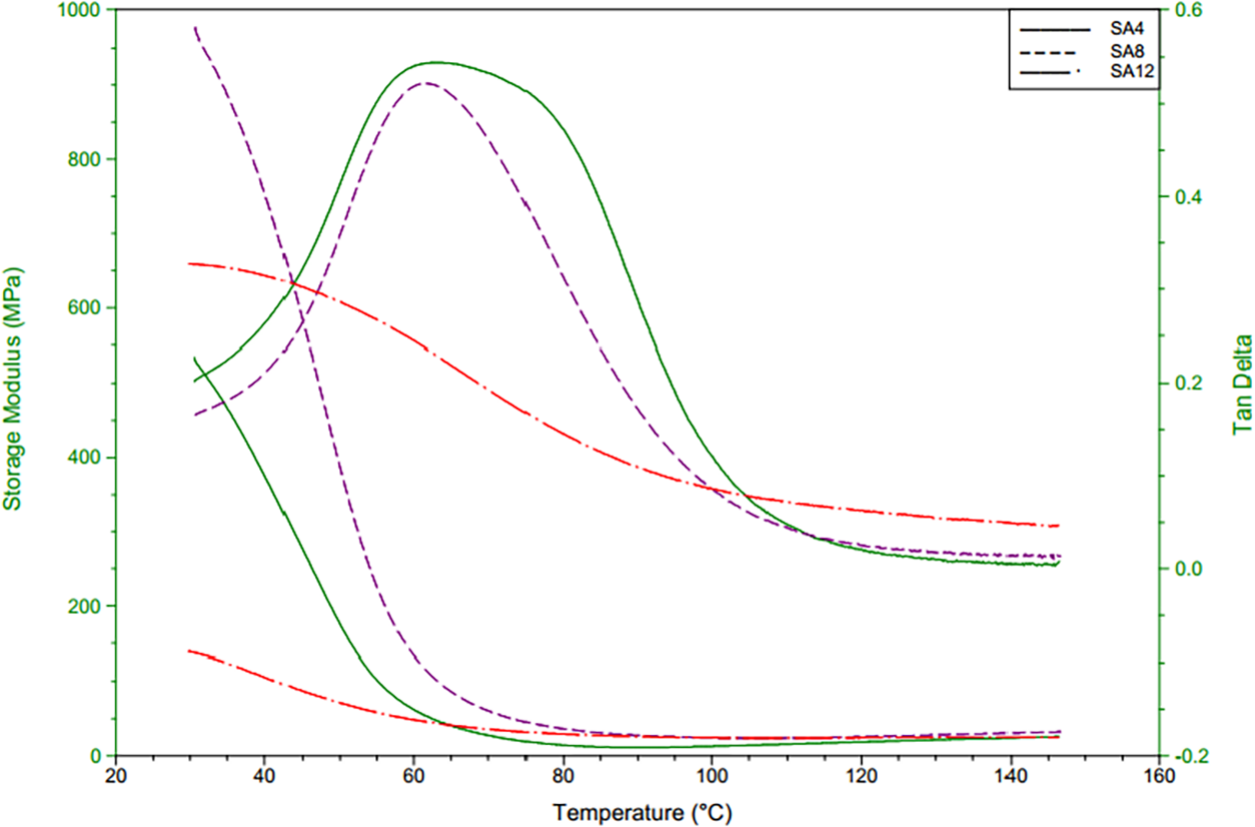
Storage modulus (*E*′) vs tan delta as a function of temperature for selected samples obtained from DMA analysis. Set A samples have higher modulus values, reflecting a more rigid network.

**Table 2:** Dynamic Mechanical Properties of Different Samples

composition	sample name	starch% (W/V)	coupling agents% (W/V)	*T*_g_ °C	glassy modulus (*E*′ at 40°) MPa	rubber plateau modulus (*E*′ at 120 °C) MPa	*T*an δ peak	interpretation
EGMA–MAA–PEGDMA	SA4	0%	None	58–60	520	120	0.45	Rigid and well-defined transitioned network
	SA7	20%	None	58–62	630	150	0.48	Reinforcing effect while slightly broader relaxation with a heterogeneous network
	SA8	30%	None	62–65	900	200	0.50	Reinforcement effect, while broader relaxation suggests heterogeneity in the network
	SA12	30%	10% CIC	55–58	130	35	0.32	Reduced effective network stiffness and lower network integrity
MAA–glycerol–APC–PEGDMA	SD3	20%	10% CIC	Broad viscoelastic transition without a distinct Tg	220	16	0.2	Broad viscoelastic relaxation and a highly plasticized network
	SD4	30%	10% CIC	Broad viscoelastic transition without a distinct Tg	230	14	0.19	Broad viscoelastic and plasticized network

The thermomechanical behavior of the samples was evaluated through temperature-sweep dynamic mechanical analysis. The evaluated values included glass transition temperature (*T*_g_), storage modulus in the glassy region (*E*′ at 40 °C), rubbery plateau modulus (*E*′ at 120 °C), and damping factor (tan delta­(δ)), and these helped to assess network rigidity, cross-link density, and structural homogeneity.

For Set A samples, a well-defined thermoset-like behavior was found in the materials. The resin with no reinforcement (SA4) showed a clear glass transition at around 58–60 °C, while the glassy modulus was in the higher region around 520 MPa, along with a relatively high rubbery modulus of around 120 MPa, indicating the formation of a rigid cross-linked network. However, the incorporation of starch showed an increase in the stiffness of the material, with 20% loading (SA7) resulting in glassy modulus increasing to around 630 MPa and the rubbery modulus around 150 MPa while maintaining a similar *T*_g_ range. This indicates the reinforcing effect of starch without major disruption of the general architecture. Increasing the loading to 30% (SA8) increased the modulus further, around 900 and 200 MPa, respectively, indicating a restricted chain mobility due to filler–matrix interactions. However, the increased tan δ peak, along with a slight broadening of the transition, indicates the growing network heterogeneity caused by polymer–starch phase separation. On the other hand, the addition of the coupling agent CIC (SA12) significantly affected the network architecture; as a result, *T*_g_ decreased to 55–58 °C while both glassy and rubbery modulus values dropped drastically to around 130 and 35 MPa, respectively. The reduced plateau modulus indicates a decrease in effective cross-link density, which suggests that the coupling agent caused a disruption in the hydrogen-bonded network and its newly formed bonds acted more as a plasticizing compatibilizer rather than reinforcing an interfacial bonding in this system.^88–91^

The Set D networks showed slightly different results than Set A, as both SD3 and SD4 samples showed no distinct *T*_g_ while also displaying low rubbery modulus values of around 14–16 MPa with low tan δ peaks of 0.19–0.20 as well. The absence of a sharp glass transition indicates a broad relaxation spectrum characteristic of highly plasticized, flexible, and loosely cross-linked networks. Increasing the starch content from 20% to 30% increased the stiffness very slightly but did not significantly change the viscoelastic nature of the material, indicating the significant dominance of polyol plasticization over reinforcement effects. The material behaves more closely to a soft elastomer or viscoelastic structure rather than a conventional thermoset.^92,93^

The combination of high gel fraction with a low rubbery modulus found in polyol-based systems suggests that chemical network formation does not necessarily translate into mechanical stiffness of the samples, highlighting the importance of interpreting the gel fraction alongside viscoelastic measurements.

Therefore, the overall effect can be described in this way: Set A is a rigid thermoset with a high modulus, clear *T*_g_, and high cross-link density, and Set D possesses a plasticized viscoelastic network architecture with no clear *T*_g_ and low modulus. Starch loading primarily acts as a reinforcing filler in rigid networks but behaves differently as a diluent in highly plasticized and flexible systems. Coupling agents reduced cross-link density by disrupting the initially formed hydrogen-bonding network in all of the samples.

### 3.4 Gel Fraction Test Results

Figure 4 shows the gel fraction test results of several samples from the combinations.

**Figure 4:**
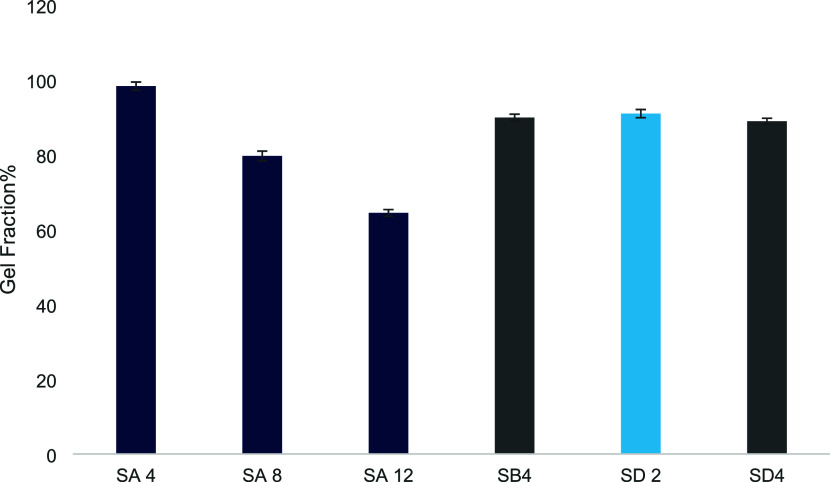
Gel fraction of the selected samples after solvent extraction. Set A samples show higher gel fraction values, indicating greater network continuity, whereas the starch loading and coupling treatment result in a reduction in gel fraction, suggesting increased extractable contents.

Gel fraction measurements could help assess the degree of cross-linking and solvent stability of the photocured samples. The gel content represents the insoluble portion of the polymer in a specific solvent after solvent extraction and therefore reflects the effectiveness of cross-linking in network formation. The assessment can be evaluated in this way: high gel fraction (>95%) as highly cross-linked thermoset networks, moderate gel fraction (80–90%) as flexible but continuous networks, and lower gel fraction (<70%) as a disrupted network due to filler interference.^94–96^

The Set A system showed the highest gel fraction among all formulations, with sample SA4 exhibiting nearly complete network formation (∼98%), confirming efficient radical cross-linking and formation of a tightly bound three-dimensional network. However, incorporation of starch reduced the gel fraction, with SA8 showing a moderate decrease to 79% and SA12 exhibiting a significant drop to 64% with the addition of the coupling agent CIC. The reductions indicate the interference of starch with polymer chain propagation while creating nonreactive domains, resulting in lower cross-link density and increased extractable fractions, which increased further with the addition of CIC, disrupting the hydrogen-bonding network and forming new bonds that may reduce homogeneity in the network, forcing phase separation and reducing contents in the gel fraction test.

The polyol-based sets B and D showed a moderately high gel fraction around 90%, suggesting that, even though the material behaves mechanically as a highly plasticized system, network formation still occurs, possibly by hydrogen bonding. The relatively high gel content combined with soft mechanical behavior implies the formation of a flexible but chemically continuous network rather than a weakly bonded polymerized structure.^89,94,96^

In contrast to polyol-based samples, which maintain a relatively high gel fraction but exhibit low rubbery modulus (chemically continuous but mechanically flexible networks), samples from set A exhibit both high gel fraction and high rubbery modulus (rigid cross-linked networks). These results demonstrate that effective reinforcement depends not only on insoluble network formation but also on the stiffness and homogeneity of that network.

### 3.5 Thermogravimetric Properties (TGA) of the Photocured Samples

Thermal properties were measured using TGA to determine the decomposition temperature of weight losses of 5%, 10%, and 50%. The results are listed in Figure 5.

**Figure 5:**
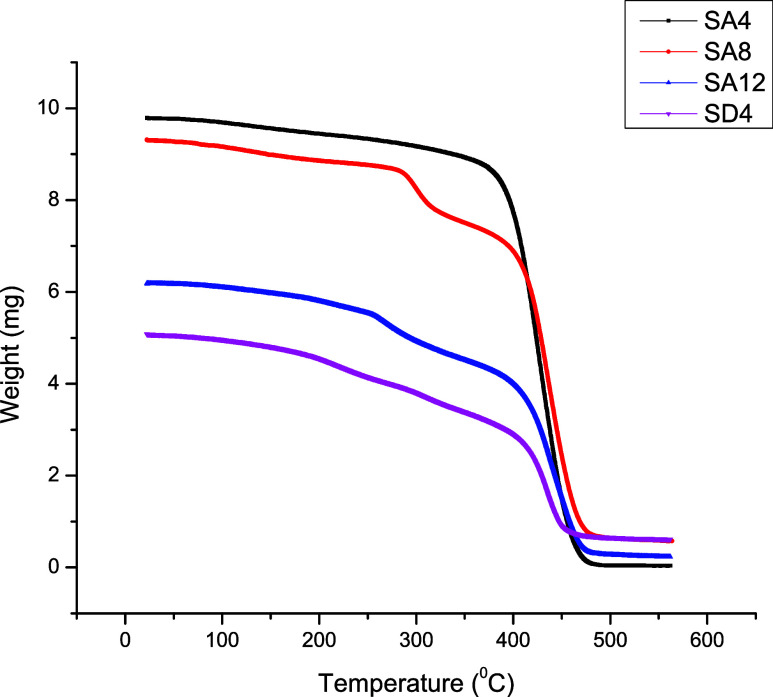
Thermogravimetric analysis (TGA) curves of selected samples demonstrating the weight loss as a function of temperature. Differences in degradation behavior as a function of temperature highlight the influence of resin composition and starch loading on thermal stability.

The first step of 5% weight loss is rarely related to the main cycle of degradation, as this portion is lost due to moisture or other similar components evaporating from the material. The latter weight losses are more reflective of material properties. The TGA results show that the addition of starch increased the degradation temperature slightly, although the impact is almost negligible, while forming more chars in all sets of samples. This occurred due to the comparatively higher thermal resistance of starch found in the previous report.^97^ While the structures containing coupling agents showed slightly better results in Set A, possibly due to the reduced mobility in the structure due to the newly introduced compounds and their bonds, which helped to slightly increase resistance to degradation.^98–100^ Set D showed the poorest results due to APC in the structure, which is known to have poor thermal resistance.^101,102^

### 3.6 Analysis of the Chemical Structure by FTIR for Different Combinations

Figure 6 shows the FTIR spectra of different samples from various sets containing 10% CIC and 30% starch, while the spectrum of MAA is also included. MAA is an acid, and its peak corresponding to the C=O stretching of carboxylic acid groups is around 1689 cm^–1^. All these samples use the coupling agent CIC for ester linking. Absorption bands in the range 1680–1750 cm^–1^ correspond to carbonyl stretching vibrations, and peaks near ∼1700 to 1720 cm^–1^ indicate contributions from acid and ester groups.

**Figure 6:**
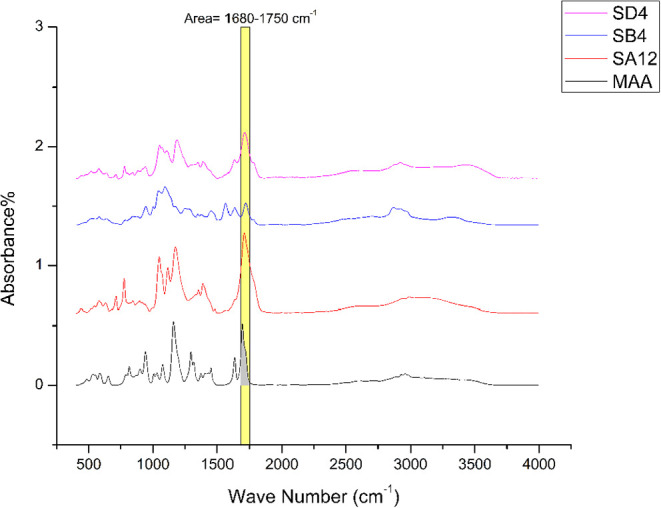
FTIR spectra of the selected samples containing 30% starch and 10% CIC, with MAA for reference. The spectra show typical carbonyl absorption bands in the 1680–1750 cm^–1^ region, corresponding to acid and ester functionalities in the system.

The presence of MAA has shifted the peaks around 1700 cm^–1^ due to its own COO peak at a lower wavelength than usual. In addition, all these ester-cured samples showed peaks around 1700 cm^–1^, which could also have resulted from the strong hydrogen-bonded structure of these materials. The presence of vinyl double bonds also has the potential to affect the results, bringing the acid peak below 1700 cm^–1^, while the ester peaks are found around 1700–1710 cm^–1^. Overall, the spectra suggest that the system contains carbonyl-containing functional groups; there may be chemical interactions between the components, but FTIR cannot definitively confirm how much covalent bond formation has occurred.^103,104^

### 3.7 Structural Analysis by SEM for Different Combinations

The effect of starch loading on different combinations is shown in the scanning electron microscopy images in Figure 7.

**Figure 7:**
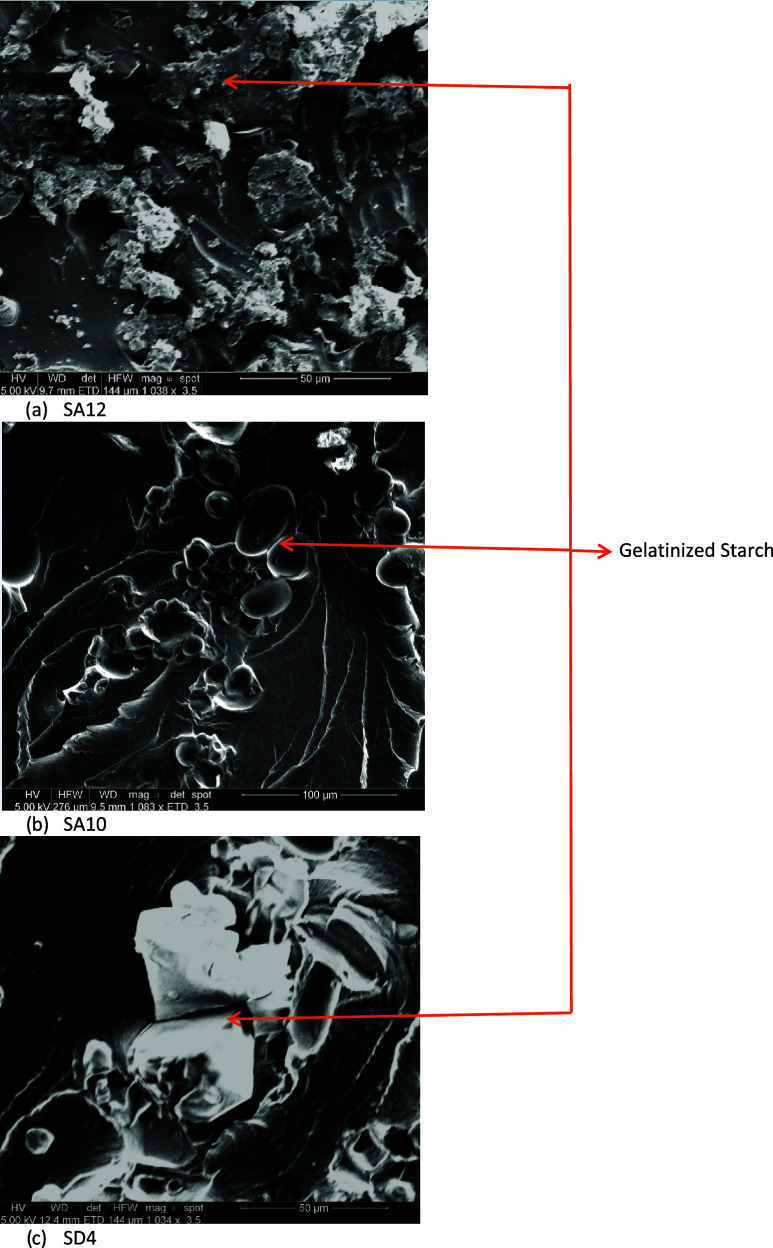
SEM images of the cross-sectional morphology of the selected samples at the same magnification. Set A samples show partially aggregated starch domains, whereas Set D samples show a more homogeneous structure, suggesting better dispersion and reduced phase separation.

Scanning electron microscopy (SEM) was used to study the morphology of the selected samples, and the cross-sectional images are presented in Figure 7. All images were taken at the same magnification for comparison. The morphology of Set A samples (SA12), which contain 30% starch, shown in Figure 7a, exhibits irregular and partially deformed starch domains, suggesting incomplete dispersion and the formation of aggregated regions within the matrix, possibly due to poor compatibility between the starch phase and the surrounding polymer network. Figure 7b,c compares Set A (SA10) and Set D (SD4) samples, where the morphology of the latter appeared more collapsed and less defined, suggesting an improved dispersion and a more homogeneous morphology. The morphology differences imply that the resin composition has a significant influence on the starch distribution within the matrix. The more uniform structure observed in Set D is consistent with better interaction between starch and the surrounding matrix, which is in agreement with the lower phase separation observed in the PSI results. The fracture surfaces also present nonuniform crack propagation, which is consistent with a ductile-type failure behavior in these materials.^85,105–111^

Therefore, these images indicate the potential swelling, gelatinization, and plasticization of starch, while the presence of acidic compounds potentially helped the process as well. The images also showed that the composites were not broken in a uniform manner, indicating the ductile nature of the materials studied here.

### 3.8 Structure–Property Relationship

The findings collectively show that the reinforcing role of starch in water-free photocurable networks is determined by resin chemistry; methacrylate-based systems produced higher strength, higher storage modulus, and clearer thermoset-like transitions, suggesting the formation of a rigid network, while starch acted as a reinforcing phase and also induced higher phase separation at higher loading; polyol-based systems, on the other hand, produced better dispersion and lower phase separation but lower mechanical strength and rubbery modulus, suggesting plasticized and more flexible networks. Therefore, improved compatibility did not produce a high mechanical performance. The results show that compatibility and stiffness are traded off: systems rich in methacrylate produce stronger networks, while systems rich in polyol improve starch dispersion but decrease the load-bearing capacity.

## 4 Conclusion

This study was conducted to develop starch-reinforced photocurable composite structures using lower-toxicity polymer systems with water-free processing conditions. The results showed that resin composition can play an important role in starch dispersion, network structure, and performance. The EGMA–MAA–PEGDMA system achieved the highest mechanical performance (highest tensile strength), with starch acting mainly as a reinforcing phase. In contrast, more biobased polyol-based systems showed improved compatibility and dispersion but reduced mechanical strength (due to a more flexible network structure). The addition of starch improved mechanical properties and increased phase instability at higher loadings (as reflected by PSI results). The coupling agents did not improve performance but rather disrupted the network uniformity by altering key interactions that were responsible for starch compatibility. The results from DMA and gel fraction indicated that the methacrylate-based systems formed more rigid networks, and hydroxyl-rich systems resulted in more heterogeneous but better starch-compatible structures.

When combined, these studies show that resin chemistry controls starch dispersion, network architecture, and reinforcement efficiency in photocurable materials. Methacrylate-rich formulations improve mechanical performance but increase phase heterogeneity, whereas polyol-based formulations are more compatible but produce weaker plasticized networks. This study demonstrates that when creating water-free biopolymer-containing photocurable materials, compatibility, cross-link density, and phase stability should be taken into account.

## Data Availability

The data underlying this study are available in the published article.
